# Single-molecule analysis uncovers the difference between the kinetics of DNA decatenation by bacterial topoisomerases I and III

**DOI:** 10.1093/nar/gku785

**Published:** 2014-09-17

**Authors:** Ksenia Terekhova, John F. Marko, Alfonso Mondragón

**Affiliations:** 1 Department of Molecular Biosciences, Northwestern University, Evanston, IL 60208, USA; 2 Department of Physics and Astronomy, Northwestern University, Evanston, IL 60208, USA

## Abstract

*Escherichia coli* topoisomerases I and III can decatenate double-stranded DNA (dsDNA) molecules containing single-stranded DNA regions or nicks as well as relax negatively supercoiled DNA. Although the proteins share a mechanism of action and have similar structures, they participate in different cellular processes. Whereas topoisomerase III is a more efficient decatenase than topoisomerase I, the opposite is true for DNA relaxation. In order to investigate the differences in the mechanism of these two prototypical type IA topoisomerases, we studied DNA decatenation at the single-molecule level using braids of intact dsDNA and nicked dsDNA with bulges. We found that neither protein decatenates an intact DNA braid. In contrast, both enzymes exhibited robust decatenation activity on DNA braids with a bulge. The experiments reveal that a main difference between the unbraiding mechanisms of these topoisomerases lies in the pauses between decatenation cycles. Shorter pauses for topoisomerase III result in a higher decatenation rate. In addition, topoisomerase III shows a strong dependence on the crossover angle of the DNA strands. These real-time observations reveal the kinetic characteristics of the decatenation mechanism and help explain the differences between their activities.

## INTRODUCTION

DNA topoisomerases are molecular machines that alter DNA topology and are found in all domains of life (prokaryotes, archaea, eukaryotes, and even some viruses) (1,2). Depending upon whether a transient single- or double-stranded break is formed in the DNA phosphodiester backbone, topoisomerases are classified as either type I or type II enzymes (1). Further classification into subclasses within each type is based upon shared sequence and structural characteristics. Type IA topoisomerases change DNA topology by creating a single-stranded break in the DNA via a 5′ phosphotyrosine linkage, followed by passage of a single or double strand of DNA through the break (3). This mechanism has been termed ‘enzyme-bridged strand passage’ as it requires the opening of a DNA gate mediated by conformational changes in the enzyme. An atomic model for the enzyme-bridged strand passage mechanism was initially proposed based on structural and biochemical results (4–6) and was further supported by single-molecule studies (7,8). Enzymes belonging to the type IA subclass are capable of relaxing negatively supercoiled DNA (1,3), and also of catenating and decatenating double-stranded DNA (dsDNA) circles and knotting and unknotting dsDNA molecules provided they have a single-stranded region (9). In the case of decatenation/catenation, the presence of a nick in a dsDNA molecules is sufficient for activity (9).

*Escherichia coli* topoisomerases I and III are the members of the type IA class and are structurally similar proteins, sharing a common toroidal core consisting of four domains that form a positively charged cavity capable of accommodating either single-stranded DNA (ssDNA) or dsDNA inside the central hole (6,10). The C-terminal domain of each protein is different. The C-terminus of topoisomerase I contains a zinc-binding domain (11–14) that facilitates DNA binding and is essential for the relaxation of negatively supercoiled DNA (13). In contrast, topoisomerase III has a short C-terminal domain that has been found to be dispensable for activity (11,12). In addition, topoisomerase III has a loop at the base of the central hole, termed the decatenation loop, which is essential for the decatenation activity of topoisomerase III (10,15).

The main cellular functions of these two related topoisomerases are distinct. The ability to decatenate DNA molecules, but not to relax DNA, was identified as a main cellular function of topoisomerase III, whereas DNA relaxation, but not decatenation, is mainly carried out by topoisomerase I (16,17). Thus, topoisomerase I is mainly engaged in relaxing the excessive number of negative supercoils that arise during transcription and in maintaining the appropriate level of DNA supercoiling in the cell (18,19).

By contrast, bacterial topoisomerase III and its closely related relative, eukaryotic topoisomerase III, are known to participate in the resolution of DNA Holliday junctions (20–23), intertwined ssDNA structures that arise during recombination. One particularly interesting role of topoisomerase III, both in bacteria and in higher organisms, is to resolve double Holliday junctions through the concerted action of topoisomerase III and a RecQ-family helicase (20,23). The helicase unwinds the duplex and provides an ssDNA substrate for topoisomerase III which unlinks the DNA strands. In this manner, topoisomerase III, a type IA enzyme, can unlink duplex DNA molecules by cleaving one DNA strand at a time. The reaction has to proceed through a hemicatenated intermediate as the reaction necessitates two strand cleavage and passage events through a single-stranded region.

In addition, decatenation of replicating daughter chromosomes by *E. coli* topoisomerase III was demonstrated *in vivo* (24,25), and interlinked ssDNAs, which resulted from the conversion of replication forks, were also suggested as a target of topoisomerase III action (26). The oriC DNA replication system, which forms very similar substrates to replication and recombination DNA intermediates, was reconstituted *in vitro* to compare decatenation activities of *E. coli* topoisomerase I and topoisomerase III in bulk experiments (27,28). These experiments showed clearly that whereas topoisomerase III has robust decatenation activity, topoisomerase I is almost unable to separate catenated molecules (24–26). It was estimated that *in vitro E. coli* topoisomerase III decatenates interlinked dsDNA circles with a single-stranded region about 500 times more efficiently than *E. coli* topoisomerase I (15). Furthermore, it was shown that due to its decatenation activity, topoisomerase III plays an active role in chromosomal segregation during cell division (24,26,29).

All these results suggest that in the cell topoisomerases I and III have complementary functions, with topoisomerase III being able to resolve not only ssDNA structures similar to DNA Holliday junctions but also dsDNA substrates with ssDNA regions. *In vitro* experiments have also demonstrated that the two topoisomerases display complementary activities: topoisomerase I relaxes supercoiled DNA efficiently but decatenates DNA molecules poorly, whereas topoisomerase III clearly favors decatenation (16,28).

Recent single-molecule studies of DNA relaxation found that topoisomerase I is more efficient at relaxing supercoiled DNA than topoisomerase III, in agreement with bulk experiments (30). These experiments rationalized the differences in the relaxation mechanism of the two enzymes and revealed that shorter pauses between relaxation events allow for an overall more efficient DNA relaxation reaction by topoisomerase I, despite topoisomerase III being more efficient at relaxing DNA in individual runs. Thus, the overall relaxation rate is a combination of two main characteristics, pause length and relaxation rate per run. The relaxation rate per run is slower for topoisomerase I than topoisomerase III, but the pause length is much longer for topoisomerase III with the result that topoisomerase I is overall much more efficient at relaxing DNA than topoisomerase III. Finally, single-molecule DNA decatenation studies using braided DNA molecules with a single-stranded DNA gap showed that topoisomerase III can decatenate these molecules efficiently, but that topoisomerase I cannot act on them (31). The experiments show that topoisomerase III acts very efficiently on gapped substrates, which are similar to the ones used in bulk experiments (15), but surprisingly topoisomerase I cannot decatenate these substrates. From these experiments, a rate of ∼5 catenanes/s was obtained for topoisomerase III.

The single-molecule observations on DNA relaxation provide an explanation for the differences in behavior by topoisomerases I and III that could not be concluded from bulk experiments. However, while they provide a solid explanation for the differences in relaxation between the two enzymes, the previous single-molecule experiments do not help to understand the differences in decatenation activity, the other major reaction catalyzed by the two enzymes. In order to understand the mechanistic differences in the activity of these two prototypical type IA topoisomerases, we analyzed DNA decatenation activity at the single-molecule level. We used DNA braids formed by dsDNA with an ssDNA region to mimic catenated DNA at the single-molecule level. We were able to detect real-time decatenation of two dsDNA molecules by *E. coli* topoisomerase I and topoisomerase III. Topoisomerase III was more efficient at decatenation than topoisomerase I, consistent with *in vivo* and bulk observations. However, the additional kinetic information obtained from our single-molecule experimental observations provides new insights into the mechanism of action of these enzymes, illuminating their distinct roles in the cell.

## MATERIALS AND METHODS

### Enzymes and DNA substrates

**Figure 1.**
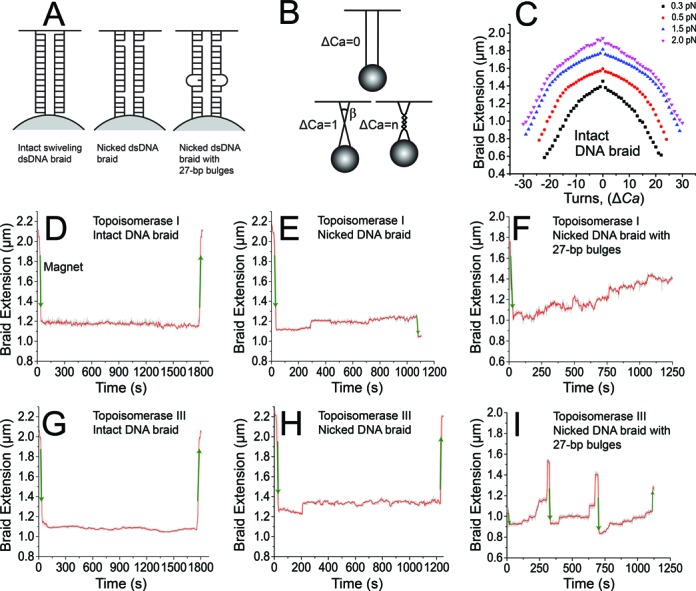
Decatenation of DNA braids by *E. coli* topoisomerases I and III. (**A**) Sketch of two DNA molecules attached to a paramagnetic bead: two intact dsDNAs attached by only one strand (left), two nicked dsDNAs (middle) and two nicked dsDNA with a 27-bp bulge (right) on each DNA molecule. The three substrates were braided by rotating the magnet and were used in *E. coli* topoisomerases I and III decatenation experiments. These DNA braids mimic catenated DNA structures that arise during chromosomal replication and catenated dimers of nicked circular DNA. (**B**) Diagram illustrating the braiding procedure and the crossover angle (β) between two braided strands. The crossover angle is obtained by braiding two parallel DNA molecules (top) by one turn (Δ*Ca*) (bottom left). A more catenated DNA substrate is formed by braiding two DNA molecules by several turns (Δ*Ca* = *n*) (bottom right). (**C**) Example of an extension versus catenation number for a nicked DNA braid with 27-bp bulges at different forces (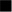: 0.3 ± 0.1 pN; 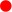: 0.5 ± 0.15 pN; 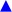: 1.5 ± 0.2 pN; 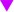: 2.0 ± 0.3 pN). (**D–I**) Plots showing typical decatenation runs for different substrates. Each run is defined as a series of decatenation events in which pausing cannot be observed. (D) Decatenation of an intact dsDNA braid by topoisomerase I. No activity was observed for this type of substrate. (E) Decatenation of a nicked dsDNA braid by topoisomerase I. Poor activity was observed with this substrate, confirming that the presence of the nicks is not sufficient to recapitulate robust decatenation activity. (F) Decatenation of a nicked dsDNA braid with a 27-bp bulge by topoisomerase I. (G) Decatenation of an intact dsDNA braid by topoisomerase III. (H) Decatenation of a nicked dsDNA braid by topoisomerase III. (I) Decatenation of a nicked dsDNA braid with a 27-bp bulge by topoisomerase III. In all cases, the length of the DNA is plotted against time. Manual introduction or removal of the catenanes resulted in shortening or elongation, respectively, of the DNA braid, (green arrows), whereas decatenation by an enzyme resulted in elongation. The gray trace corresponds to the measured events, whereas the red trace corresponds to an unweighted running average over 50 events.

*E. coli* topoisomerases I and III were purified as described elsewhere (4,32). For the experiments, three types of DNA molecules were prepared: intact DNA, nicked dsDNA and nicked dsDNA with a 27-bp bulge, where a bulge refers to an unpaired ‘extra’ string of nucleotides introduced in only one strand (Figure 1A). The DNA molecules had biotin (bio) and digoxigenin (dig) functionalized ends to use as attachments and were made as described in (30). For the single-molecule experiments, two DNA molecules were attached from the dig-labeled ends to a glass slide functionalized with anti-dig antibodies, whereas the other ends were attached to a streptavidin-coated paramagnetic bead (Invitrogen) through the biotin-labeled ends (33). An amount of 0.4 attomol of functionalized DNA in 2 μl of phosphate buffer saline was incubated with 6 μl of 1-μm-diameter paramagnetic beads (3 ng/μl) in 0.4-mg/ml bovine serum albumin (BSA) for 12 min with gentle agitation at room temperature followed by 10-min incubation with the glass slide.

The first type of DNA used, a braid of two identical intact dsDNA molecules, corresponds to a catenated substrate without any nicks or ssDNA regions. In this case, only one DNA strand from each dsDNA was attached to the bead and the glass, leaving the other strand free to rotate. This makes it possible for the two intact DNAs to braid but not to supercoil. The 6.2-kb dsDNA molecules were derived from the pFOS-1 plasmid and were created by polymerase chain reaction amplification utilizing 28-bp primers containing bio-labels and 26-bp primers containing a 5′-end dig-label (34). The dig primer had two digoxigenin molecules, at the 5′-end and one internal, whereas the bio primer had only one 5′-end label.

The second type of braid studied corresponds to two nicked braided substrates, while the third type corresponds to two braided nicked dsDNAs with a 27-bp bulge on each DNA molecule. These three types of braids will be referred to as ‘intact’, ‘nicked’ and ‘bulge’ hereafter.

The 7.3-kb DNA molecules with bio- and dig-functionalized ends were prepared in identical ways as described in (30). For the decatenation experiments, four nicks 1103 bp and 1469 bp from each end were introduced into each dsDNA by Nb.BsrDI nickase (New England Biolabs, MA). The nicks are located 2622 bp and 2256 bp from the bulge on the dig-end and 1902 bp and 1536 bp from the bulge on the bio-end. The nicks permit swiveling of the tethered dsDNAs, thus preventing the accumulation of torsional stress inside the dsDNAs as they are wound around one another.

### Experimental decatenation procedures: DNA selection, DNA manipulation and protein introduction

The magnetic tweezers setup used for the experiments has been described elsewhere (35,36). The stretching force applied to the DNA was controlled by altering the distance between the bead and the magnets. The catenation state of DNA, which is described by the catenation number *Ca*, was controlled by rotation of the magnets. Data were collected at 50 Hz, which allowed us to resolve single decatenation events in most cases. When very fast decatenation bursts (two to three events) were observed, single events could not be resolved. Force calibration was carried out using analysis of the bead fluctuations following the method in (33). All experiments were done at 37°C using a heater to control the temperature of the chamber.

Selection of DNA molecules suitable for decatenation experiments was conducted based on DNA catenation (magnet rotation) versus DNA extension plots. Plots were obtained for each type of DNA substrate; these plots reflected the dependence of the DNA braid extension upon the magnet rotation for a number of forces in the 0.3–2-pN range (Figure 1C and Supplementary Figure S1). Braids containing two nicked (or single-strand-attached) DNA molecules produced a well-characterized, nearly left–right symmetric extension versus *Ca* curve in a rotation versus extension plot (34), while braids containing double-strand-tethered molecules that were not nicked produced left–right asymmetric extension versus *Ca* curves due to the introduction of superhelicity in the DNA. In addition, acceptable DNA braid candidates displayed a similar relaxed extension value (within 0.1 μm) and exhibited similar length changes upon catenation in the same force regime (Figure 1C and Supplementary Figure S1). The DNA extension versus *Ca* curves also served to directly relate the bead displacement in the z- or length-direction to the change in catenation number.

After finding appropriate doubly-dsDNA-tethered beads, 30–35 turns were introduced into the braid. Protein was injected by flowing in 400 μl of reaction mixture containing 2 nM of either *E. coli* topoisomerase I or topoisomerase III in 50-mM Tris-HCl pH 8.0, 120-mM NaCl, 1-mM MgCl_2_ and 200-μg/ml BSA. If no decatenation activity was observed 30 min after the first of injection, an additional 200 μl of enzyme mixture was introduced. This procedure was repeated until decatenation was observed. Once decatenation was observed, the activity of topoisomerase I or III was monitored until DNA decatenation had ceased for 10 min, after which the DNA was braided again via the magnets. If 45 min after braiding the DNA decatenation was not observed, 2 nM of protein in 200-μl volume was injected, thus replacing the protein solution.

**Figure 2.**
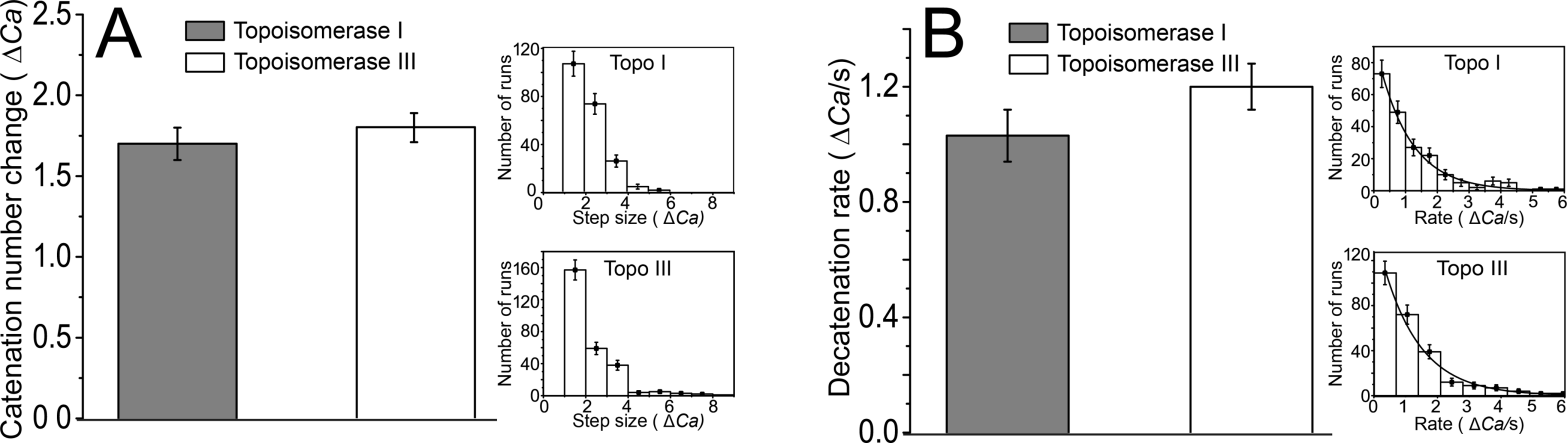
Topoisomerases I and III have similar number of decatenation events and decatenation rates per run. (**A**) The number of catenanes per run resolved by topoisomerases I and III on nicked DNA braids with 27-bp bulges. Histogram of the mean number of catenanes resolved in a run (Δ*Ca*). The two enzymes remove similar numbers of catenanes in a run. The inset shows the distribution for topoisomerase I (top) and topoisomerase III (bottom). The distributions do not fit to a simple exponential decay and hence the average number of turns removed was calculated. (**B**) Decatenation rate per run by topoisomerases I and III on nicked DNA braids with 27-bp bulges. The histogram shows the distribution of the decatenation rate per run. Topoisomerase III has a similar decatenation rate per run as topoisomerase I. The inset shows the distribution for topoisomerase I (top) and topoisomerase III (bottom). The solid curve corresponds to a fit of an exponential decay to the distribution. In both panels, shaded bars correspond to topoisomerase I and white ones to topoisomerase III and error bars correspond to the standard error of the mean. Details on the number of events used for each histogram are found in Supplementary Table S1.

**Figure 3.**
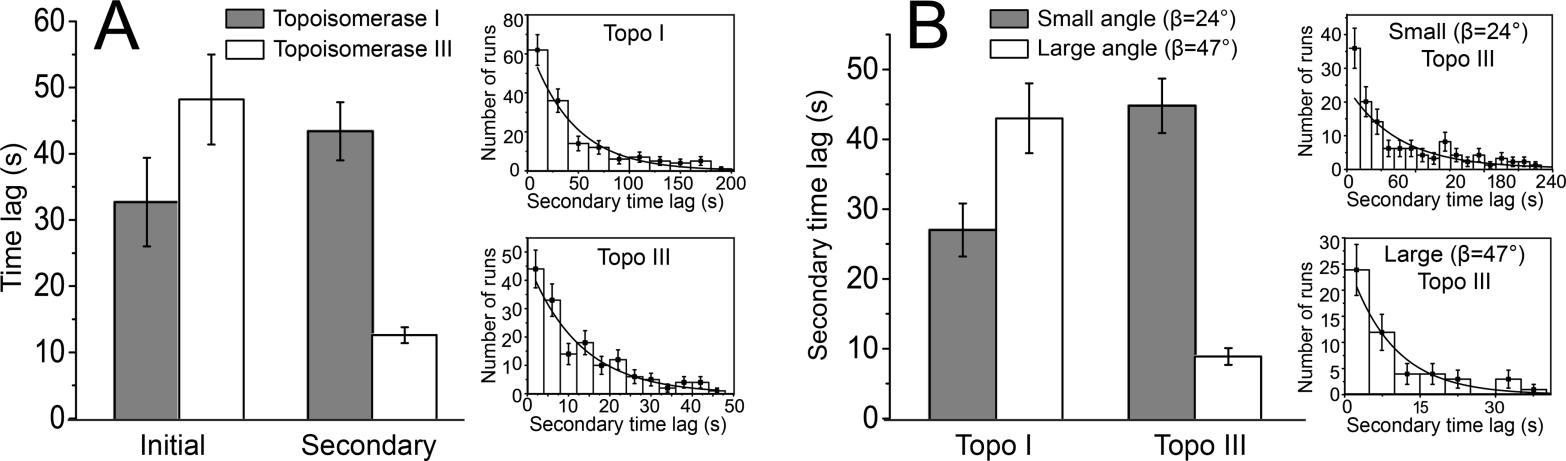
Time lags before a decatenation run for topoisomerases I and III. (**A**) Initial and secondary time lags for topoisomerases I and III on nicked DNA braids with 27-bp bulges. Histogram of initial time lag before decatenation indicates that topoisomerases I and III are comparable. In contrast, histogram of the secondary time lag indicates a larger difference between the two enzymes (*P*-value < 0.0001). The inset shows the distribution of secondary time lags for topoisomerase I (top) and topoisomerase III (bottom). (**B**) Effect of crossover geometry of the DNA braid on the secondary time lag by topoisomerases I and III. Histogram showing the distribution of secondary time lag for two sets of DNA crossover angles for topoisomerase III. Topoisomerase III exhibits shorter secondary time lags in the case of large crossover angles (*P*-value < 0.0001), whereas topoisomerase I has similar secondary time lags for all crossover angles (not shown). Shaded bars correspond to small (β = 24°) and white to large (β = 47°) angles (see the text for a definition of the crossover angle). The inset shows the distribution of secondary time lags for topoisomerase III activity for small (top) and large (bottom) crossover angles. In all histograms, the error bars correspond to the standard error of the mean. The solid curve corresponds to a fit of an exponential decay to the distribution. Details on the number of events used for each histogram are found in Supplementary Table S1.

**Figure 4.**
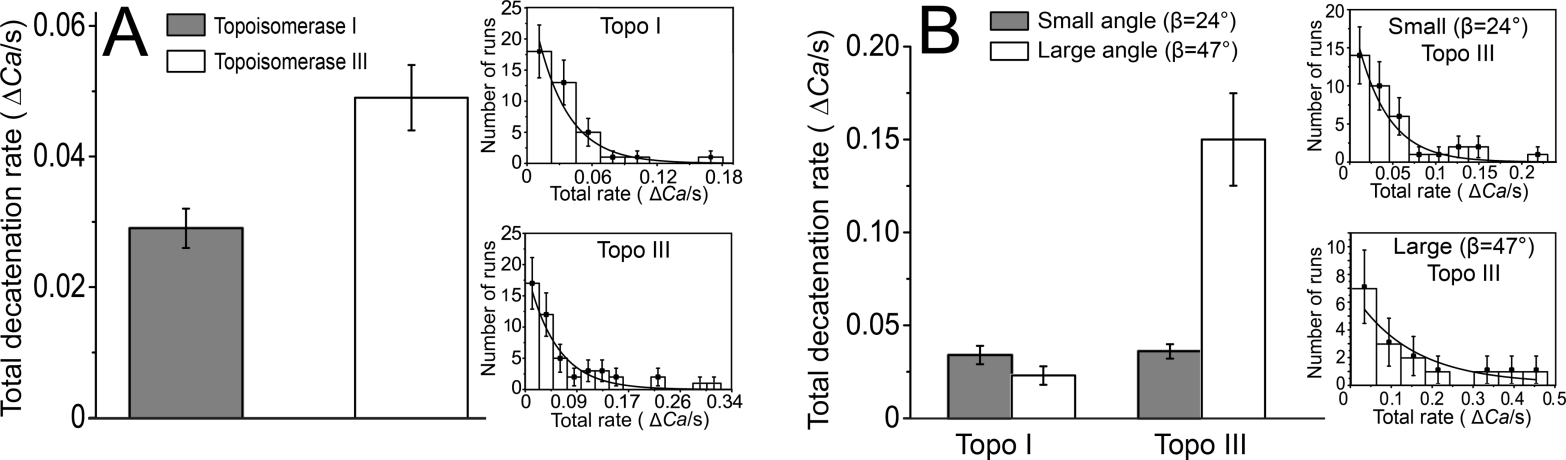
Topoisomerase III is a faster decatenating enzyme. (**A**) Total decatenation rate by topoisomerases I and III on a nicked DNA braid with 27-bp bulges. Histogram shows the distribution of the total decatenation rate. Topoisomerase III has a significant (*P*-value < 0.0001) faster total decatenation rate than topoisomerase I, in agreement with bulk experiments. The inset shows the distribution of total decatenation rates for topoisomerases I (top) and III (bottom). (**B**) Effect of braid geometry on the total decatenation rate by topoisomerases I and III. Histogram showing the distribution of the total decatenation rate for two DNA crossover angles. It indicates that topoisomerase III has a larger total decatenation rate for large crossover angle, whereas the decatenation activity of topoisomerase I is not strongly dependent on the crossover angle. Shaded bars correspond to small (β = 24°) and white to large (β = 47°) angles (see the text for a definition of the crossover angle). The histogram of the total decatenation rate indicates a significant difference between the two crossover angles in the case of topoisomerase III (*P*-value < 0.0001). The inset shows the distribution of total decatenation rate for topoisomerase III for small (β = 24°) (top) and large (β = 47°) (bottom) crossover angles. In all histograms, the error bars correspond to the standard error of the mean. The solid curve corresponds to a fit of an exponential decay to the distribution. Details on the number of events used for each histogram are found in Supplementary Table S1.

Decatenation events were analyzed by measuring five different features: initial time lag, secondary time lag, number of catenanes released per run, decatenation rate per run and total decatenation rate (Supplementary Figure S2). Regions of interest were selected manually from recordings of bead displacement versus time and processed with a custom-written Matlab script (MATLAB 6.1, The MathWorks Inc., Natick, MA). Data analyses were performed with Matlab (www.mathworks.com) and Origin Pro (www.originlab.com). Data for each of the parameters were binned into histograms (Figures 2–4). The mean values (t1) for each of the parameters (Tables 1 and 2) were derived from fits of an exponential decay function (***Ae*^−*tx*^**) to the histograms of the experimental values (Origin, OriginLab, Northampton, MA). In the histograms, the errors were calculated as the standard deviation of the number of observations. The errors for the mean values correspond to the standard errors. The number of events observed for each parameter is shown in Supplementary Table S1. Supplementary Table S3 shows the values for the fits of all the parameters analyzed. To assess the significance of the differences in the mean values of the parameters, the *P*-value was calculated from a Student's *t*-test. For more detailed information on data analyses, see the Supplementary Data section.

**Table 1.** Mean value of the parameters for the characterization of the decatenation of dsDNA braids with 27-bp bulges by *E. coli* topoisomerases I and III

Parameter	Enzyme	
	Topoisomerase I	Topoisomerase III
Initial time lag (s)	33 ± 8	48 ± 8
Secondary time lag (s)	43 ± 5	13 ± 1
Catenanes removed (Δ*Ca*)	1.7 ± 0.1*	1.8 ± 0.1*
Decatenation rate per run (Δ*Ca*/s)	1.0 ± 0.09	1.2 ± 0.08
Total decatenation rate (Δ*Ca*/s)	0.029 ± 0.005	0.049 ± 0.01

The table shows the mean values for the five parameters used to characterize the single-molecule DNA decatenation events. In all cases, the mean, either computed from the fit of an exponential decay function or as an arithmetic average (marked by an asterisk), and the fitting error or the standard error of the mean are shown.

**Table 2.** Dependence of the decatenation of dsDNAs with 27-bp bulges on the DNA crossover angle

Parameter	Topoisomerase I		Topoisomerase III	
	Crossover angle		Crossover angle	
	Small β	Large β	Small β	Large β
Initial time lag (s)	53 ± 7 *	54 ± 6*	41 ± 7 *	41 ± 7*
Secondary time lag (s)	27 ± 4	43 ± 8	45 ± 7	9 ± 2
Catenanes removed (Δ*Ca*)	1.7 ± 0.1	1.7 ± 0.1	1.9 ± 0.1	1.7 ± 0.1
Decatenation rate per run (Δ*Ca*/s)	1.0 ± 0.08	1.0 ± 0.08	0.9 ± 0.09	1.1 ± 0.25
Total decatenation rate (Δ*Ca*/s)	0.034 ± 0.005*	0.023 ± 0.005*	0.036 ± 0.008	0.15 ± 0.04

For topoisomerase I, the decatenation reaction characteristics are largely independent of the crossover angle. For topoisomerase III, significant differences are observed in the secondary time lags and the total rate of decatenation. For topoisomerase III, an increase in the crossover angle leads to a decrease in the pauses between the decatenation runs and an increase in the total rate of DNA decatenation. In all cases, the mean, either computed from the fit of an exponential decay function or as an arithmetic average (marked by an asterisk), and the fitting error or the standard error of the mean are shown.

## RESULTS

To characterize the decatenation activities of *E. coli* topoisomerases I and III at the single-molecule level and to address the possible mechanisms leading to chromosomal segregation by topoisomerase III, we used three types of dsDNA substrates. In all cases, we used two braided DNA molecules to mimic two catenated dsDNA. The first type, two braided intact dsDNA molecules, should not be a substrate for a type IA topoisomerase as there are no ssDNA regions for the protein to act on. The second substrate, two braided dsDNA molecules containing a bulge, should present the best substrate as the bulge in the DNA should serve as a region for a type IA enzyme to work on. The braided molecules are expected to be topologically similar to replicated chromosomal DNAs encountered by topoisomerases in the cell. The third type, two braided nicked dsDNA molecules, were used to ensure that the nicks present in the bulged molecules were not responsible for the decatenation activity. The bulged molecules were nicked to prevent supercoiling of the DNA during braiding. The nicks are far from the bulges but in principle could act as short gapped regions to permit decatenation. It was thus important to show that the decatenation level due to the nicks was very low.

As type IA topoisomerase alters the topology of DNA using an enzyme-bridged strand passage mechanism, it is impossible to decatenate two dsDNA molecules in a single step. Supplementary Figure S3 shows a schematic representation of the possible steps that must take place for the decatenation of two dsDNA molecules with a single-stranded bulge by a type IA topoisomerase. For the release of one catenane, two DNA cleavages and two protein opening and closing events must occur and a hemicatenated intermediate is required. One of the cleavages must be in the bulge region and leads to the hemicatenated intermediate; the second one has to occur in another region in the complementary strand. Cleavages only in the single-stranded bulges would not lead to decatenation of the molecules. For these reasons, release of one catenane corresponds to two catalytic cycles of the enzyme involving two strand passage events and a hemicatenated intermediate.

### Selection of braided substrates

To form the braids, two DNA molecules were attached to a bead using biotin-labeled ends and to the glass surface using the dig-labeled ends (see the Materials and Methods section) and braided manually by rotating the magnet (Figure 1B). To prevent supercoil formation during the braiding process, the individual DNA molecules were either nicked or were attached using only one of the two strands, thus allowing swiveling (Figure 1A). For the braid relaxation experiments, only positively braided DNA substrates were analyzed. We chose positively braided DNA for our experiments as during DNA replication and enzyme-based recombination the catenated molecules created are right-handed (37) and these were some of the cellular events that we seek to recapitulate in our experiment. A limited number of experiments with negatively braided DNA substrates were done and showed similar trends, but there were not enough observations to do a quantitative comparison with the positive braids. DNA braids have been well characterized before and are easily recognizable by the shape of the curve in an extension versus *Ca* plot, which is symmetric, has a sharp peak at zero turns and has a slope that does not change substantially with force (38). In contrast, the extension versus *Ca* plot for a single DNA molecule is not left–right symmetric, varies smoothly and has a strongly force-dependent slope (33).

All experiments on the three types of braids were performed at 2.0-pN force (estimated error ± 0.3 pN). The choice of this force value was influenced by the force regime previously used in relaxation experiments on a supercoiled DNA substrate with a 27-bp bulge (30). A force of 2.0 pN is favorable due to strong suppression of thermal motion-driven movements of the bead and also due to stabilization of the ssDNA bulge.

### Qualitative decatenation behavior by topoisomerases I and III

In a typical decatenation experiment, a single DNA braid was formed by introducing 30–35 positive turns, which led to a length decrease, and then was subjected to decatenation by the enzyme, resulting in length increases. Clear pauses between the intertwining and the first decatenation event and also in between runs of decatenation events were observed. Once the braid was fully decatenated, or at least 10 min after a decatenation run was observed, it was catenated again. This procedure was repeated several times per pair of DNA strands, until the DNA braid became damaged (displayed an irreversible change in its extension versus *Ca* response) or detached from the glass.

Figure 1D–I show examples of real-time measurements of DNA decatenation events by *E. coli* topoisomerases I and III. As expected, no activity by topoisomerase I or topoisomerase III was detected on braids formed by intact dsDNA molecules (Figure 1D and G). Both enzymes exhibited robust decatenation activity on nicked dsDNA braids with 27-bp bulges, as shown in Figure 1F and I. In contrast, decatenation activity by both topoisomerases was observed only in ∼10% of the experiments on nicked dsDNA braids, even after waiting for ∼90 min (Figure 1E and H), showing that the presence of nicks is not enough to recapitulate the robust activity observed with the bulged molecules and supporting the use of nicked, bulged molecules for the experiments.

### Quantitative analysis of decatenation kinetics and crossing geometry

In order to analyze and characterize the decatenation events and to determine the differences between the two enzymes, five characteristic features of the DNA decatenation events on the bulged DNA braid were analyzed: (i) initial time lag, (ii) secondary time lag, (iii) number of catenanes released per run, (iv) decatenation rate per run and (v) total decatenation rate (Supplementary Figure S2). A decatenation run represents a series of decatenation events that are closely spaced in time and cannot be resolved into individual decatenation events by our instrument due to a combination of the finite sampling rate ∼40–50 Hz and Brownian noise. In the majority of the cases, the events were clearly resolved by the instrument, but there were few instances where possible pauses between two decatenation events could not be resolved. In many instances, a decatenation run consists of a single catenane release (Δ*Ca* = 1), but sometimes the runs consists of several closely spaced events (Δ*Ca* = *n*). The initial time lag refers to the time between the formation of the initial DNA braid and the first decatenation run detected. The secondary time lag refers to the time between two decatenation runs, discounting initial runs. The decatenation rate per run refers to the number of catenanes released in a run divided by the time spanned. Finally, the total decatenation rate refers to the total number of catenanes released divided by the time from the moment the braid was catenated until the braid becomes fully decatenated, as judged by it reaching its original, relaxed extension. The results of tens of decatenation observations (see Supplementary Table S1 for details on the number of observations) were analyzed by calculating these five parameters, creating histograms of the frequency of the observed values and computing the mean from the distribution of these values.

In addition, the geometry of the braids was analyzed to estimate the DNA crossover angle (β) (Figure 1B). For a given force, the DNA crossover angle is related to the height of the jump between *Ca* = 0 and *Ca* = ±1 in the extension versus *Ca* plot. The relation of the DNA crossover angle to the height of the peak is expressed by the following formula, $\beta = 2arccos\left( {\frac{{l - h}}{l}} \right)$, where *β* is the DNA crossover angle, *l* is the length of the DNA and *h* is the height of the peak at *Ca* = 0 in the extension versus *Ca* plot. This formula does not take into account the curvature of the bead, the separation of the strands or the diameter of the dsDNAs, as in other approaches (38,39). It corresponds to the formula of Charvin *et al.* (38) when the diameter of the strands is zero and is adequate for the purpose of classifying the braids into broad groups based on the crossover angle. The DNA crossover angles were classified into two groups, small or large angle groups. The two groups were selected by noticing a bimodal distribution of the angles and each angle represents the mean of each group. All members of a group were within 10° of the mean value. All the events used for calculating the overall values were used and separated into two groups. A few events that did not fit well into one or the other group were discarded. A breakdown of the number of events used for the overall and the crossover angle calculations is given in Supplementary Table S1. For example, for topoisomerase I in only one instance was an angle observed whose value was larger than 10° from the mean and this observation was discarded. Thus, the mean of the two groups is: small angle *β* = 24° ± 3° for topoisomerase I and topoisomerase III and large angle *β* = 49° ± 1°/*β* = 47° ± 2° for topoisomerase I/topoisomerase III.

### Decatenation by topo I and topo III requires DNAs with nicks and bulges

The mean number of decatenations in a run by topoisomerases I and III on the braided DNA bulge substrate was found to be <Δ*Ca>* = 1.7 and <Δ*Ca>* = 1.8, respectively. (Figure 2A and Table 1). In the case of decatenation of nicked DNA braids with a 27-bp bulge, the number of catenanes released was the same for the small and large crossover angles. On a nicked DNA braid weak decatenation activity was observed in only ∼10% of the experiments, even after waiting for over 60 min after initial catenation (Supplementary Table S2), confirming that the nicks in the molecule, which are distant from the location of the bulges and the center of the molecules, are not responsible for most of the decatenation events. Decatenation was never detected on an intact DNA braid.

The initial and secondary time lags were also analyzed (e.g. initial time lags versus secondary time lags; topoisomerase I versus topoisomerase III) (Figure 3A and Table 1). For the nicked DNA substrate, the mean time lag was 3–5 min, but this is clearly an underestimate as in most cases (∼90%) no events were detected even after a long wait. In contrast, decatenation activity by both topoisomerase III and topoisomerase I on a nicked catenated DNA substrate with 27-bp bulges was always detected within less than 1 min after initial braiding. These observations suggest that both topoisomerase I and topoisomerase III can occasionally act through nicks on the catenated substrates, but for robust levels of decatenation activity they require longer ssDNA regions located near the center of the molecules, the expected location of the braids, as has been observed before (31).

### Topoisomerase III pauses for a shorter time between successive decatenation bursts than topoisomerase I

In the case of a catenated DNA substrate with 27-bp bulges, both time lags had a distribution that could be fit by a simple exponential decay, and from these distributions it was possible to extract the mean decatenation reaction time (Figure 3). The initial time lags of decatenation were comparable for topoisomerase I and topoisomerase III, 33 ± 7 s and 48 ± 7 s, respectively (Table 1 and Figure 3A). In contrast, the secondary time lags were different and much shorter for topoisomerase III, 13 ± 1 s, compared to 43 ± 4 s for topoisomerase I (Table 1 and Figure 3A). In other words, the pauses between decatenation runs catalyzed by topoisomerase I are 3.4 times longer than for topoisomerase III. In addition, the mean time lags for topoisomerase I were similar regardless of whether they were for primary or secondary events.

### Topoisomerase III displays a strong dependence of pauses between decatenation events on dsDNA crossing angle

In order to determine the dependence of the pauses between decatenation events on the DNA crossover angle, exponential fitting to the histogram of the distributions of values allowed computation of the mean values for the different parameters. Interestingly, the secondary time lags for topoisomerase III exhibited a strong dependency upon the crossover angle of the catenated DNA substrate with the 27-bp bulge. In the case of topoisomerase III, doubling of the crossover angle led to a 5-fold decrease in the length of the pauses between decatenation runs (Figure 3B and Table 2), whereas for topoisomerase I the effect was more modest and the pauses were longer for the larger angles. Notably, the initial time lags and decatenation rate do not depend strongly upon the DNA crossover angle value (Table 2). The change in the secondary pause length due to the change in geometry of the braids supports the idea that the secondary time lags are not waiting times for binding events, as could be the case for the initial time lags, but pauses of an enzyme already in place. Overall, larger crossover angles of the DNA strands lead to shorter pauses between the decatenation runs for topoisomerase III, whereas the crossover angle does not have a marked effect on topoisomerase I waiting times.

We also analyzed the decatenation rate per run, i.e. the number of catenanes removed per second in a run (Table 1 and Figure 2B). Topoisomerase I and topoisomerase III have the same decatenation rate per run, removing 1 catenane/s on the nicked DNA braid with a 27-bp bulge. These rates do not depend on the crossover angle of the DNA strands (Table 2). Finally, the overall decatenation rate takes into account the pauses between runs and is a quantity that is directly comparable to the results from bulk experiments. Analysis of the overall decatenation rate reveals that topoisomerase III is faster in overall DNA decatenation rate than topoisomerase I on the bulged substrate (Table 1 and Figure 4B), even though the decatenation rate per run is similar for both enzymes (Figure 2B). Analysis of the crossover angle effect on total decatenation rate showed that for topoisomerase III doubling the angle results in a 4-fold increase in the overall decatenation rate, whereas it does not have a large effect on topoisomerase I (Table 2 and Figure 4B). The decatenation activity is highly dependent on the presence of a single-stranded region. The decatenation rate for both topoisomerase I and topoisomerase III on the nicked catenated substrate without a ssDNA region is close to zero.

Table 1 shows the mean values for the five characteristics analyzed for decatenation by topoisomerase I and topoisomerase III of two positively braided intact DNAs, two nicked dsDNAs without bulges and two nicked dsDNA with ssDNA bulges. Comparing these values, the single-molecule decatenation experiments recapitulate the bulk experiments well; topoisomerases I and III do not act on dsDNA. On a nicked catenated substrate with ssDNA bulges, the total decatenation rate for topoisomerase III, 0.05 ± 0.005 Δ*Ca*/s, is larger than the total decatenation rate for topoisomerase I, 0.03 ± 0.003 Δ*Ca*/s, in agreement with the bulk results that show that topoisomerase III is a better decatenating enzyme. The enhanced decatenation activity of topoisomerase III is associated with shorter secondary time lags and is also dependent on the substrate geometry. Catenated DNA strands with large crossover angles are even more favorable for topoisomerase III (0.15 ± 0.02 Δ*Ca*/s) than for topoisomerase I (0.02 ± 0.005 Δ*Ca*/s), suggesting that an ideal substrate for topoisomerase III includes a large crossover angle.

Overall, the results from the experiments can be summarized as follows: topoisomerase I and topoisomerase III resolve catenated DNA with ssDNA regions with similar rates per run as well as similar waiting times for the first decatenation run. However, after the first decatenation run the pauses between subsequent decatenations events are much shorter for topoisomerase III. This combination of kinetic characteristics results in topoisomerase III having a slightly faster total decatenation rate than topoisomerase I and leads to more efficient decatenation activity by topoisomerase III. In addition, the geometry of the braids is important for topoisomerase III; upon an increase in the DNA strand crossover angle the pauses between the decatenations runs become shorter, whereas topoisomerase I activity is insensitive to such changes.

## DISCUSSION

In bulk experiments, type IA topoisomerases can decatenate DNA molecules provided one of them has a nick or an ssDNA region (9). From these experiments, it was estimated that topoisomerase III is about 500 times more efficient than topoisomerase I in the decatenation reaction (15). Recent single-molecule experiments also show that topoisomerase III can decatenate braided DNA with single-stranded gaps efficiently, although in these experiments there was no activity observed for topoisomerase I. In these experiments, the substrate used are catenated DNA molecules with single-stranded regions, quite different from the bulged DNA molecules used in the current experiments. The faster decatenation rate observed for gapped molecules in the single-molecule experiments (∼5 Δ*Ca*/s) is likely due to the very different substrates used and the fact that only one decatenation event was measured per experiment. When the decatenation rates of single runs are compared, the numbers are comparable (5 versus 1.2 Δ*Ca*/s). In addition, in the gapped DNA molecule experiments only one strand passage event is needed, decatenation of DNA molecules with a bulge requires at least two strand passage events and also the relocation of the enzyme to the second strand. Thus, the two experiments are measuring slightly different reactions of different complexity. The lack of activity by topoisomerase I for gapped substrates is still surprising, especially as this enzyme does decatenate bulged DNA braids. Nevertheless, it is important to note that the single-molecule experiments with braided DNA molecules mimicking catenated molecules reproduced the behavior observed in bulk experiments: the overall decatenation rate for topoisomerase III was higher than for topoisomerase I. The consistency in the ranking of the bulk experiments using different substrates with our single-molecule approach using braided molecules is remarkable and validates the use of bulged molecules for the study of decatenation by these topoisomerases.

The experiments were done under conditions where only one molecule is expected to act on the DNA. Previous experiments with the same two enzymes showed that 2 nM of protein is the minimal concentration needed to observe single-molecule events (30). Furthermore, only one molecule can bind to each DNA molecule as there is only one bulge region per molecule and type IA topoisomerases require single-stranded regions for activity. A topoisomerase can also use a gap or nicked region, but the experiments with nicked molecules show that the rate of events on nicked molecules is very low. Thus, it is very unlikely that the events observed are the results of more than one molecule acting on each braid as any molecules bound to DNA outside the bulge region cannot perform any topological transformations. In the case of braided nicked DNA molecules, the important consideration is the presence of a single-stranded regions; the absence of such a region makes it impervious to action by a type IA topoisomerase.

The primary and secondary time lags observed for topoisomerase I are comparable (33 s versus 43 s) and could represent waiting times for the enzyme to bind. For topoisomerase I, it is impossible to tell whether the secondary time lags are different from the time waiting for a molecule to find the bulge region. The binding affinity of *E. coli* topoisomerases I and III for short single-stranded DNA is ∼200 and ∼500 nM (4,32), respectively. The protein in the experiments is at much lower concentration than the *K*_d_ and hence it is possible that at higher enzyme concentrations the lag times, if they represent waiting times for binding events, could be different. In the case of topoisomerase III, the secondary time lags are considerably shorter than the primary time lags. The difference is statistically significant (48 s versus 13 s, *P*-value < 0.0001). Furthermore, using the values from the exponential decay that fit the data distributions it is possible to estimate the fraction of events that would overlap. The data are fitted well by a simple exponential decay (Supplementary Table S3) and there is no evidence of two processes with different decay constants, as would be expected if the secondary time lags represented binding times and another different type of event. For the secondary time lags, after 29 s, 90% of the events have occurred. For the primary time lag, 29 s is long enough for 55% of the events to occur. It is clear that the two types of lag times are different and are likely to represent two different processes. Thus, for topoisomerase III the shortness of the secondary time lags compared to the initial ones indicates that the secondary events are predominantly pauses before initiation of a decatenation event, and not waiting times for binding events as may be the case for topoisomerase I.

As expected, intact DNA braids were not decatenated by either topoisomerase I or topoisomerase III, even after a long wait. On the other hand, both enzymes were efficient at decatenation provided that the catenated DNA substrate contained ssDNA regions, such as bulges, whereas decatenation of nicked DNA braids was poor and infrequent. In fact, in the latter case only in ∼10% of the nicked DNA braids was there some activity detected and it was limited to a few decatenation events. For this reason, DNA decatenation on this type of substrate could not be analyzed thoroughly. The unbraiding experiments of a DNA braid with 27-bp bulges revealed that the two proteins decatenate DNA differently but have several similar kinetic characteristics (Table 1). For both proteins, the rate of an individual decatenation run was ∼1 catenanes removed per second (Figure 2B) and both have comparable initial lag times, but once the first decatenation run occurred, topoisomerase III was capable of initiating subsequent decatenation events much faster than topoisomerase I, on average around three times faster (13 versus 43 *Ca*/s).

Interestingly, the value of the crossover angle of the DNA braid has a large effect on the length of the pauses between decatenation events for topoisomerase III, but not for topoisomerase I. In the case of topoisomerase III, approximately doubling the crossover angle reduced the secondary lag times to ∼9 s from ∼45 s, a 5-fold decrease, whereas it did not have any effect on the other kinetic characteristics (Table 2). In the case of topoisomerase I, none of its decatenation parameters exhibited a marked dependency on the crossover angle.

The crossover angle is dependent on the separation between two attached DNAs (Figure 1B), and torque that is generated on the braid upon catenation is also related to the separation between the two DNAs (40) (torque is a function of catenation number and DNA separation (40)). Thus, variations in DNA crossover angle result in different torsional stress on a DNA braid. The shortness of the secondary time lags compared to the initial ones indicates that the secondary events are likely being performed by the same topoisomerase III molecule. It is likely that the secondary time lags are pauses before initiation of a decatenation event, and not waiting times for binding events. Accordingly, the dependence of secondary time lags on the physical characteristics of the DNA braid suggests that in the case of topoisomerase III these lags may be shorter in substrates subject to higher torque. It is possible that higher torque in the braids is more favorable for topoisomerase III as it facilitates the strand passage events. In the case of type IB and type IC topoisomerases (35,41), which relax DNA through a constrained swiveling mechanism, torque plays a major role. In that case, torque directly provides the driving force for strand swiveling. It is possible that the decatenation reaction is also dependent on torque and that torque is the driving force for the strand passage events by facilitating the movement of the DNA chains. Torque could be helping the unbraiding mechanism in a parallel manner as torque helps relaxation in swiveling topoisomerases. The relationship between torque, catenation number and crossover angle is not well understood so it is not possible to make a direct connection between torque and catenation. A better understanding of the genesis of torque in braids is necessary to make a better connection between different braid characteristics and the decatenation mechanism.

It was demonstrated previously that relaxation by topoisomerase III at the single-molecule level is very sensitive to the type of ssDNA substrate, whereas the activity of topoisomerase I is not (30). In contrast, catenated DNA substrates with a larger crossover angle, and therefore a different torque in the braid, are processed more efficiently by topoisomerase III than topoisomerase I, which shows no marked preference for the braid crossover angle. When only small crossover angle events are considered, topoisomerase III has similar overall rates to topoisomerase I. This suggests that topoisomerase III does not only favor decatenation, it favors decatenation of molecules with a large crossover angle.

The origin of this marked distinction is not apparent from the single-molecule experiments, but structures and biochemical studies of *E. coli* topoisomerase III have identified the decatenation loop as a major difference between the two closely related enzymes (10,15). The decatenation loop is at the base of the central hole and adjacent to the region that needs to separate to accept the passing strand. Moreover, recent structural and mechanistic studies of the closely related human topoisomerase IIIα in complex with a small region of RMI1 show also the presence of a crucial loop in the central hole of the protein (42). Human topoisomerase IIIα does not have a decatenation loop and it has been suggested that RMI1 provides the loop that plays a similar role to the decatenation loop in *E. coli* topoisomerase III. The role of the loop would be to bias the reaction toward decatenation in a yet undetermined manner. The observation that topoisomerase III is more efficient at decatenating substrates with a larger crossover angle also suggests that the topology of the crossover or the torque exerted by the strands may play a significant role, as discussed before. Overall, it appears that topoisomerase III behaves similarly to topoisomerase I when the substrate has a small crossover angle. This suggests that the origin of the differences resides both in the protein and the DNA and that topoisomerase III favors catenated molecules through the torque or topology created by the DNA molecules.

The present studies demonstrate that topoisomerase III is a more efficient decatenase than topoisomerase I for dsDNA braids with ssDNA regions, which represent DNA molecules with a similar topology to replicated chromosomal DNA with ssDNA regions. This decatenation activity is consistent with the role assigned to *E. coli* topoisomerase III in chromosome segregation (43), where it was shown that topoisomerase III is required for chromosome segregation and that this activity is independent of RecQ. As type IA enzymes require ssDNA regions for activity, it was suggested that topoisomerase III acts on precatenanes (43), so called as they are intermediates that if not resolved convert into catenanes (44). Thus, the present single-molecule studies are consistent with a mechanism of chromosome segregation that does not require the action of a type II topoisomerase to resolve catenanes or RecQ to unwind DNA. As long as there are single-stranded regions, topoisomerase III can efficiently decatenate the molecules. It is difficult to say what could affect the torque and/or the braid angle *in vivo* as there is no information in this regard. In the cell, processes that pull apart catenated DNA strands may affect the angle between the strands in the braid. As the two catenanes are pulled apart, the angle between the braids would increase and consequently the torque would increase. Our observations would suggest that topoisomerase III would be well suited for processes that generate tension between entangled or catenated molecules, such as mitosis and meiosis, which is in excellent agreement with the role of topoisomerase III in the cell.

Topoisomerase III can work in conjunction with a RecQ-type helicase to decatenate dsDNA molecules (20,45) or to resolve Holliday junctions (46). In this scenario, RecQ recruits topoisomerase III to an appropriate DNA substrate, which it creates by unwinding dsDNA (20,25). Additional experimental work exploiting the ability of RecQ to generate ssDNA from dsDNA will be crucial to understand the RecQ-dependent decatenation mechanisms by type IA topoisomerases. Furthermore, studying the mechanism of topoisomerase III co-acting with RecQ helicases can significantly contribute to understanding the origin of RecQ helicases-associated tumorigenesis, chromosomal instability and cancer susceptibility in humans, as well as to defining methods for their inhibition and prevention. Finally, it will be necessary to investigate topoisomerase III decatenation activity on braided ssDNAs that mimic replication and recombination intermediates containing extensive ssDNA regions or Double Holliday Junctions in order to further address their roles in the cell.

## SUPPLEMENTARY DATA

Supplementary Data are available at NAR Online.

SUPPLEMENTARY DATA
