# Supplementary material for: Single-molecule analysis uncovers the difference between the kinetics of DNA decatenation by bacterial topoisomerases I and III
Source: Nucleic Acids Res. 2014 Sep 17;42(18):11657–67. doi: 10.1093/nar/gku785 (PMC4191389; doi:10.1093/nar/gku785)
Supplement: SUPPLEMENTARY DATA [file supp_42_18_11657__index.html]

Single-molecule analysis uncovers the difference between the kinetics of DNA decatenation by bacterial topoisomerases I and III — Single-molecule analysis uncovers the difference between the kinetics of DNA decatenation by bacterial topoisomerases I and III — SUPPLEMENTARY DATA 

# Single-molecule analysis uncovers the difference between the kinetics of DNA decatenation by bacterial topoisomerases I and III

## SUPPLEMENTARY DATA

**Files in this Data Supplement:**

- SUPPLEMENTARY DATA
