# Supplementary material for: Single-molecule analysis uncovers the difference between the kinetics of DNA decatenation by bacterial topoisomerases I and III
Source: Nucleic Acids Res. 2014 Sep 17;42(18):11657–67. doi: 10.1093/nar/gku785 (PMC4191389; doi:10.1093/nar/gku785)
Supplement: SUPPLEMENTARY DATA [file supp_gku785_nar-01622-h-2014-File006.pdf]

Supplementary data

Single-molecule analysis uncovers the difference between the kinetics of DNA decatenation by bacterial topoisomerases I and III

Ksenia Terekhova[1], John F. Marko[1,2] and Alfonso Mondragón[1]

[1] Department of Molecular Biosciences, Northwestern University, Evanston, IL, 60208 USA
[2] Department of Physics and Astronomy, Northwestern University, Evanston, IL, 60208, USA.

* To whom correspondence should be addressed. Tel: +1-847-491-7726; Fax: +1-847-467-6489; Email: a-mondragon@northwestern.edu

SUPPLEMENTARY METHODS

Characterization of decatenation events: For a given force, which was manually introduced by moving the magnets, a software script permitted the calculation of five decatenation parameters from the recorded length change *vs.* time data (Figure S2). The initial time lag was estimated as the time difference between the moment when the manual DNA braiding was completed and the moment when visible protein activity occurred. The initiation and completion of protein activity on a DNA molecule were determined by manual inspection and defined as a run. This allowed us to calculate the pauses or secondary time lags between decatenation runs. The change in catenation number was determined by matching the length change to the corresponding catenation number change in the DNA catenation *vs.* extension plots at a given force. A characterization curve was obtained for each DNA substrate studied at the force used in the experiment and represented the dependence of DNA braid length on magnet rotation (Figure S1). The decatenation rate per run was calculated by dividing the change in catenation number (ΔCa) over the time interval of the run. Total rate per event was computed as a ratio of the total number of catenanes released over the total time for this event, including the initial time lag.

Parameter	Enzyme	
	**Topoisomerase I**	**Topoisomerase III**
Initial time lag (s)	40	53
Secondary time lag (s)	157	153
Catenates removed (Δ*Ca*)	207	270
Decatenation rate per run (Δ*Ca*/s)	208	137
Total decatenation rate (Δ*Ca*/s)	41	53
Total decatenation rate by crossover angle (Δ*Ca*/s)	21 Small Angle<br>14 Large Angle	36 Small angle<br>16 Large angle

Supplementary Table S1. Total number of measurements for each decatenation parameter for the DNA with 27bp bulges substrate. For the analyses of different crossover angles, the total number of events was broken into two groups, small and large crossover angle. A few events that did not agree with the criteria for dividing the angles were excluded. Details on the division of angles are given in the **RESULTS** section.

**Parameter**	**Enzyme**	
	**Topoisomerase I**	**Topoisomerase III**
Time lag (s)	337 ± 80	172 ± 50
Catenanes removed (ΔCa)	1.2 ± 0.2	1.1 ± 0.1
Decatenation rate per run (ΔCa/s)	0.2 ± 0.02	0.6 ± 0.05
Total decatenation rate (ΔCa/s)	0.003 ± 0.0004	0.006 ± 0.0005
Number of events	7	8

Supplementary Table S2. Mean value of the parameters for the decatenation of dsDNA braids with nicks by *E. coli* topoisomerases I and III. The table shows the average values for the four parameters used to characterize the single-molecule DNA decatenation events as well as the number of events observed. For dsDNA braids with nicks, only in ~10% of the experiments was decatenation observed so the parameters represent an overestimate.

Enzyme	Parameter	A	Standard fit error	t_0	Standard fit error	Reduced χ^2	Adjusted R^2	Table
**Topoisomerase I**	Number of catenates removed per run	499.003	175.198	1.039<br>1.7	0.1627<br>0.1	4.762	0.876	I
	Number of catenates removed per run for small DNA crossover angles	313.01	100.323	1.109<br>1.7	0.166<br>0.1	2.788	0.910	II
	Number of catenates removed per run for large DNA crossover angles	*	*	*<br>1.7	*<br>0.1	*	*	II
	Initial time lag	23.133	7.733	32.727	8.303	1.187	0.441	I
	Initial time lag for small DNA crossover angles	*	*	*<br>53	*<br>7	*	*	II
	Initial time lag for large DNA crossover angles	*	*	*<br>54	*<br>6	*	*	II
	Secondary time lag	66.69	9.777	43.360	4.968	1.193	0.88	I
	Secondary time lag for small DNA crossover angles	32.142	6.423	27.028	4.164	1.379	0.566	II
	Secondary time lag for large DNA crossover angles	21.191	4.92	43.091	8.175	0.753	0.687	II
	Decatenation rate per run	94.823	10.685	1.032	0.087	1.095	0.906	I
	Decatenation rate per run for small DNA crossover angles	68.062	7.195	0.984	0.0774	0.696	0.949	II
	Decatenation rate per run for large DNA crossover angles	26.182	9.143	0.605<br>1.0	0.168<br>0.1	1.962	0.640	II
	Total decatenation rate	29.156	6.818	0.029	0.005	0.863	0.792	I
	Total decatenation rate for small DNA crossover angles	*	*	*<br>0.034	*<br>0.005	*	*	II
	Total decatenation rate for large DNA crossover angles	*	*	*<br>0.023	*<br>0.005	*	*	II
**Topoisomerase III**	Number of catenates decatenated per run	678.508	153.516	1.029<br>1.2	0.103<br>0.1	2.263	0.926	I
	Number of catenates removed per run for small DNA crossover angles	407.084	146.839	0.996<br>1.9	0.158<br>0.1	2.931	0.834	II
	Number of catenates removed per run for large DNA crossover angles	153.162	29.818	1.141<br>1.7	0.107<br>0.1	0.481	0.966	II

	Initial time lag	41.573	8.742	48.194	7.874	0.81	0.849	I
	Initial time lag for small DNA crossover angles	17.094	2.896	57.746 41	9.757 7	0.295	0.895	II
	Initial time lag for large DNA crossover angles	13.846	5.153	37.611 41	10.839 7	0.545	0.692	II
	Secondary time lag	46.748	5.782	12.645	1.298	0.914	0.9	I
	Secondary time lag for small DNA crossover angles	23.487	4.272	44.947	6.851	1.219	0.657	II
	Secondary time lag for large DNA crossover angles	27.608	7.441	8.856	2.068	1.171	0.693	II
	Decatenation rate per run	150.928	14.351	1.180	0.081	1.014	0.918	I
	Decatenation rate per run for small DNA crossover angles	66.009	9.318	0.852	0.089	1.281	0.889	II
	Decatenation rate per run for large DNA crossover angles	35.444	6.663	1.049	0.250	0.975	0.874	II
	Total decatenation rate	19.918	5.038	0.049	0.011	0.917	0.661	I
	Total decatenation rate for small DNA crossover angles	20.5189	5.602	0.036	0.008	0.975	0.622	II
	Total decatenation rate for large DNA crossover angles	6.622	1.777	0.145	0.039	0.35	0.659	II

Table S3. Parameters after fitting the data distributions to a simple exponential decay function. Data for each of the parameters were binned into histograms and the mean values (t_0) for each of the parameters were derived from fits of an exponential decay function (Ae^{-tx}) to the histograms of the experimental values using Origin (OriginLab, Northampton, MA). Fits were accepted if the Reduced χ^2 and the Adjusted R^2 were close to 1. If the fits were not accepted (blue values), the mean value of the parameters was used. In some cases, it was not possible to fit the data to an exponential decay function (*) and the mean value was used instead. Parameters where the fit was poor are shown in blue and the mean values in black. For the cases where the fit did not work, the mean values are shown in black and the parameters are replaced by an asterisk (*). The last column shows the table where each parameter is reported in the manuscript.

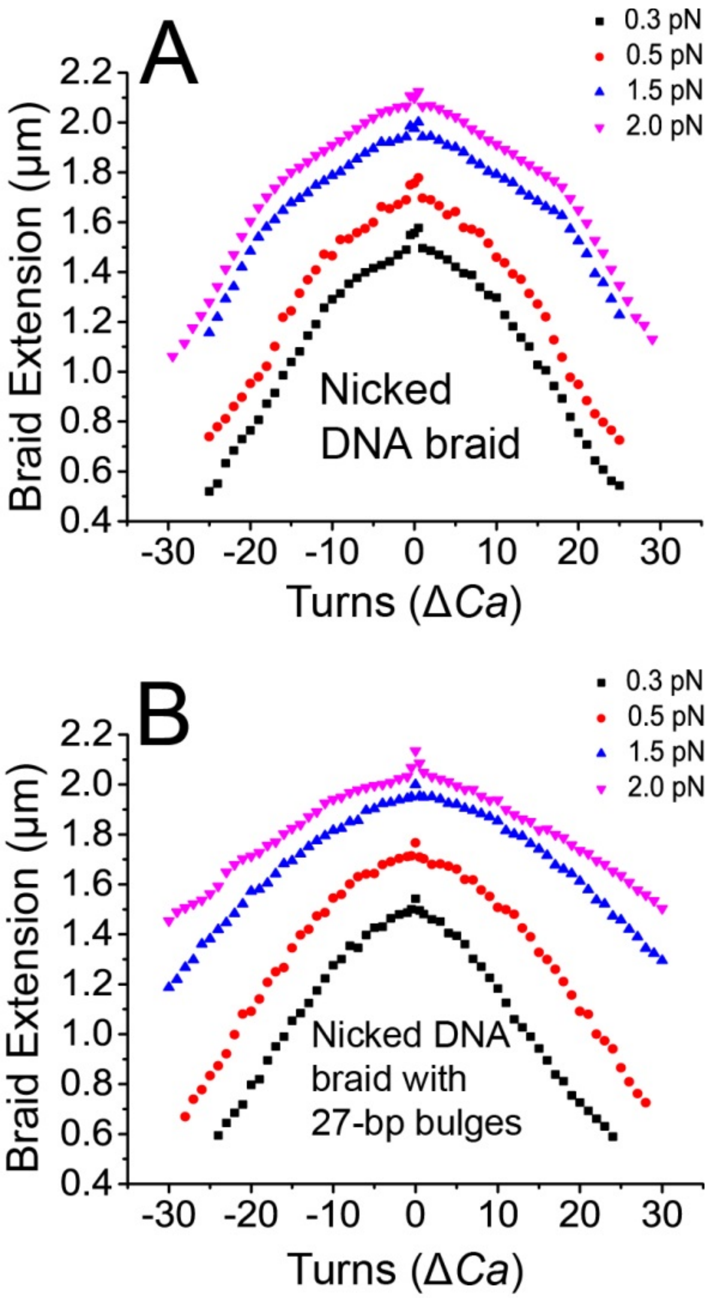

Supplementary Figure S1. DNA braid extension upon catenation at different forces for different substrates. A) Extension *vs.* catenation number for a nicked dsDNA braid. **B)** Extension *vs.* catenation number for a nicked dsDNA braid with 27-bp bulges. In both cases data were collected at different forces (■ - 0.3 ± 0.1 pN; ● - 0.5 ± 0.15 pN; ▲ - 1.5 ± 0.2 pN; ▼ - 2.0 ± 0.3 pN). Note the characteristic symmetrical shape of the plot with a sharp peak at the center.

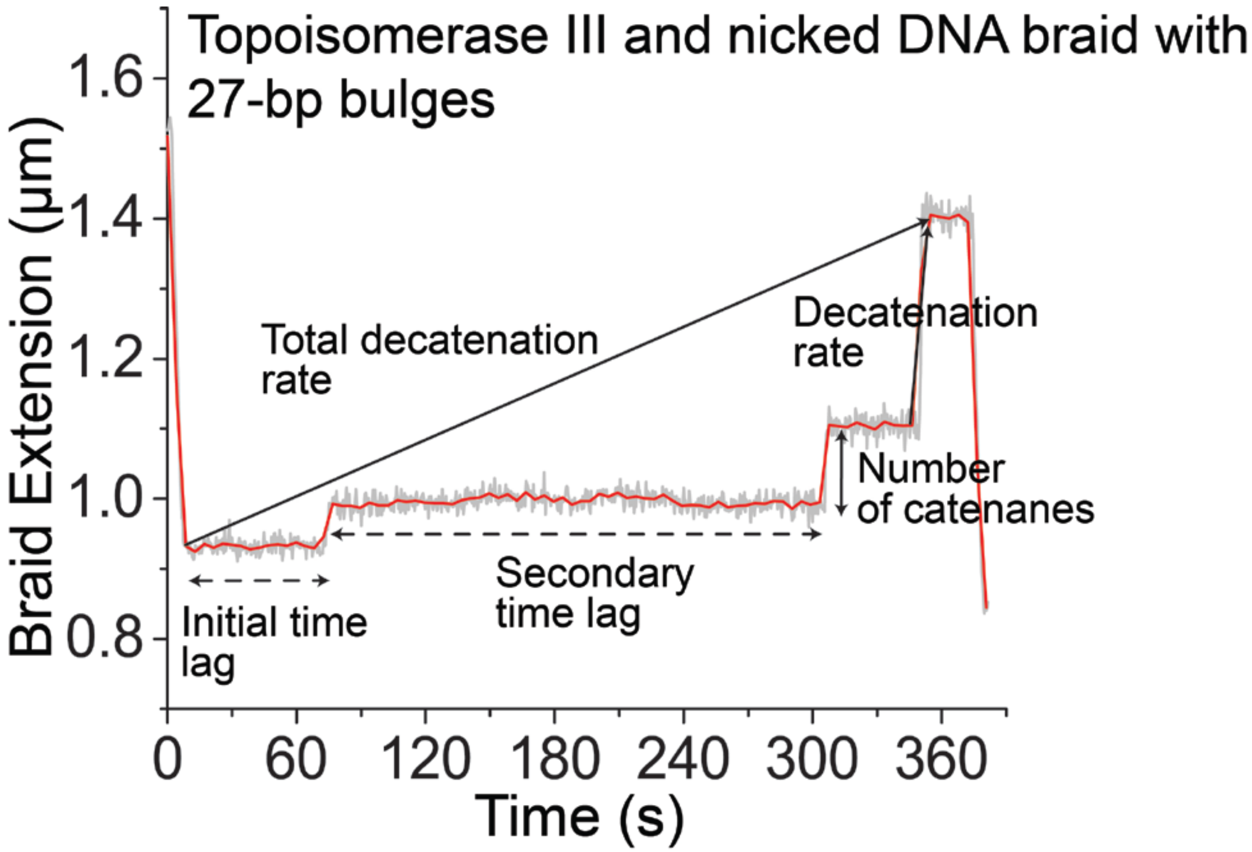

Supplementary Figure S2. Illustration of the parameters used to analyze the DNA decatenation events by topoisomerases I and III. A decatenation event between manual braiding of two DNA molecules is characterized by initial time lag (dotted line), secondary time lag (dotted line), number of catenanes released per run (solid), decatenation rate per run (solid), and total decatenation rate (solid). The gray trace shows the raw extension measurements while the red trace corresponds to an unweighted running average of the raw data over a 10-time-point window.

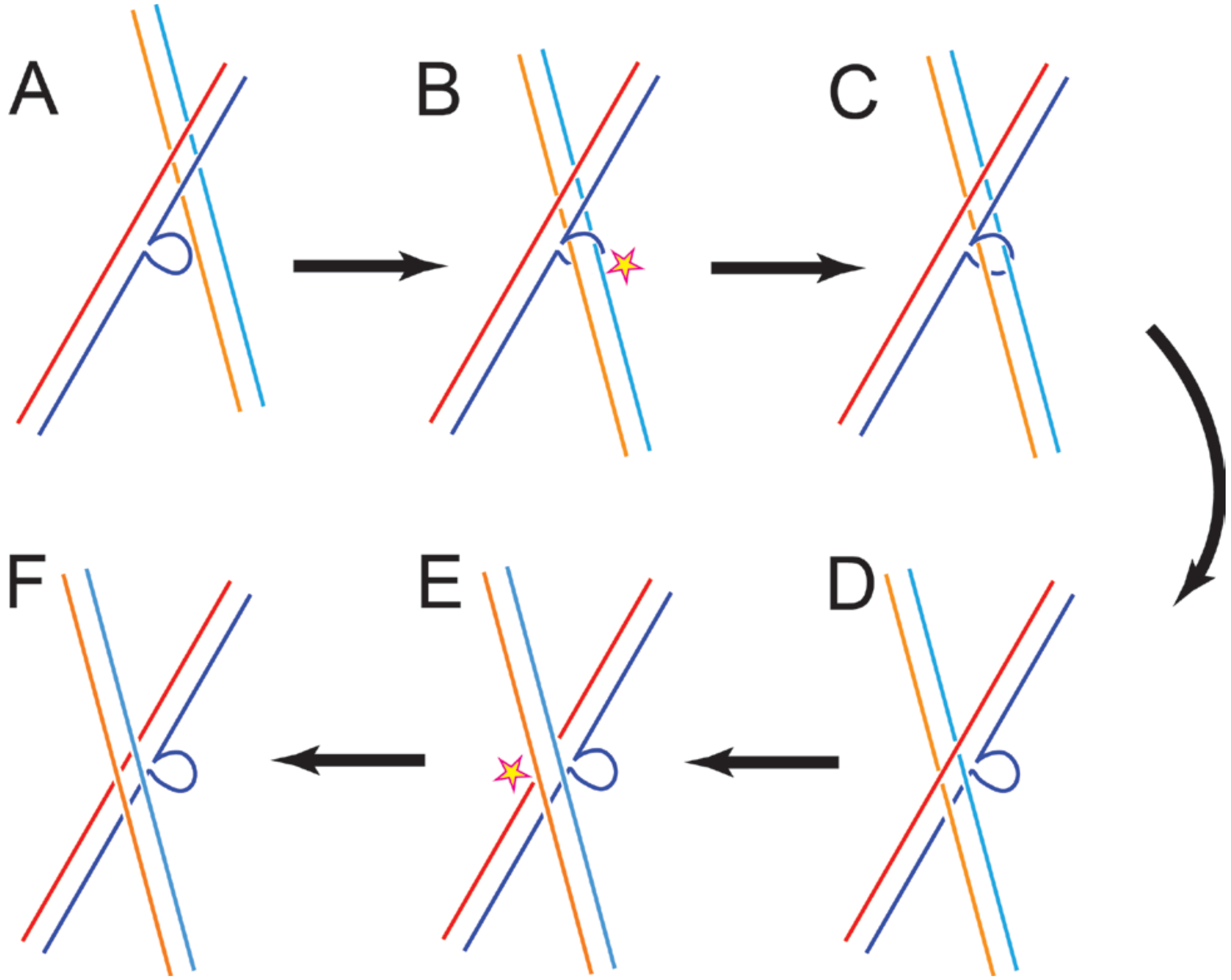

Supplementary Figure S3. Diagram illustrating a possible mechanism of decatenation of two dsDNA molecules with bulges by a type IA topoisomerase. To release one catenane in a dsDNA braid with a ssDNA bulge the passing dsDNA strand needs to enter through the bulge and then exit through the complementary strand. Thus, two cleavage and religation events are needed in each decatenation cycle and a hemicatenated intermediate is required. **A)** Braid formed by two dsDNA molecules with a ssDNA bulge. **B)** A type IA topoisomerase (star) cleaves the ssDNA bulge and creates an opening in the DNA which allows the passage of the other dsDNA through the transient break. **C)** The opening is sealed and the passing dsDNA is trapped inside the other DNA molecule. No catenane release has occurred. **D)** A hemicatenated intermediate is an obligatory requirement in this scheme. **E)** The complementary strand to the bulged strand is cleaved by a type IA topoisomerase (star) and the trapped dsDNA is released. **F)** The broken strand is religated resulting in the release of a single

catenane. According to this scheme, the release of a single catenane corresponds to two cleavage/religation cycles of a type IA topoisomerase I.
